# RETRACTION: Reactivation of p53 in cells expressing hepatitis B virus X‐protein involves p53 phosphorylation and a reduction of Hdm2

**DOI:** 10.1111/cas.70454

**Published:** 2026-06-24

## Abstract

**RETRACTION**: WangJ.‐H. , YunC. , KimS. , ChaeS. , LeeY. I. , KimW.‐H. , LeeJ.‐H. , KimW. and ChoH. , “Reactivation of p53 in cells expressing hepatitis B virus X‐protein involves p53 phosphorylation and a reduction of Hdm2,” Cancer Science 99, no. 5 (2008): 888–893, 10.1111/j.1349-7006.2008.00754.x.18294283 PMC11159080

The above article, published online on 24 February 2008 in Wiley Online Library (wileyonlinelibrary.com), has been retracted by agreement between the journal Editor‐in‐Chief, Masanori Hatakeyama; the Japanese Cancer Association; and John Wiley & Sons Australia Ltd. The retraction has been agreed following concerns raised by a third party. An investigation identified multiple instances of image duplication between the Chang and ChangX‐34 α‐tubulin bands shown in Figure 1a. Further duplications were identified between the HeLa α‐tubulin bands in Figure 1a and the Dox α‐tubulin bands in Figure 3a. In addition, the cytosol phospho‐p53 (Ser 15), Lamin B, and nuclear α‐tubulin panels in Figure 4a were found to have identical backgrounds. The authors were contacted for their comments and supporting data. Due to the time that has elapsed since publication, the data are no longer available. The authors do not agree that the western blot bands or panels were duplicated. The editors consider the results and conclusions to be unreliable. The authors H. Cho and J.‐H. Wang disagree with the retraction. The remaining authors did not respond to our notice of retraction.

**RETRACTION**: J.‐H. Wang , C. Yun , S. Kim , S. Chae , Y. I. Lee , W.‐H. Kim , J.‐H. Lee , W. Kim and H. Cho , “Reactivation of p53 in cells expressing hepatitis B virus X‐protein involves p53 phosphorylation and a reduction of Hdm2,” Cancer Science 99, no. 5 (2008): 888–893, 10.1111/j.1349-7006.2008.00754.x.18294283 PMC11159080

The above article, published online on 24 February 2008 in Wiley Online Library (wileyonlinelibrary.com), has been retracted by agreement between the journal Editor‐in‐Chief, Masanori Hatakeyama; the Japanese Cancer Association; and John Wiley & Sons Australia Ltd. The retraction has been agreed following concerns raised by a third party. An investigation identified multiple instances of image duplication between the Chang and ChangX‐34 α‐tubulin bands shown in Figure 1a. Further duplications were identified between the HeLa α‐tubulin bands in Figure 1a and the Dox α‐tubulin bands in Figure 3a. In addition, the cytosol phospho‐p53 (Ser 15), Lamin B, and nuclear α‐tubulin panels in Figure 4a were found to have identical backgrounds. The authors were contacted for their comments and supporting data. Due to the time that has elapsed since publication, the data are no longer available. The authors do not agree that the western blot bands or panels were duplicated. The editors consider the results and conclusions to be unreliable. The authors H. Cho and J.‐H. Wang disagree with the retraction. The remaining authors did not respond to our notice of retraction.

